# hAECs restore follicular development in premature ovarian insufficiency via IGFBP2/IGF1R-mediated intercellular communication

**DOI:** 10.1186/s13287-026-05064-8

**Published:** 2026-05-18

**Authors:** Wenjiao Cao, Lu Shen, Qinyu Zhang, Yating Huang, Junyan Sun, Zixin Cheng, Jing Xu, Qiuwan Zhang, Dongmei Lai

**Affiliations:** 1 International Peace Maternity and Child Health Hospital, Shanghai Key Laboratory of Embryo Original Diseases, School of Medicine, Shanghai Jiao Tong University, 910, Hengshan Road, Shanghai, 200030 China; 2 Bio-X Institutes, Key Laboratory for the Genetics of Developmental and Neuropsychiatric Disorders, Ministry of Education, Shanghai Jiao Tong University, Shanghai, 200030 China; 3 School of Biomedical Engineering, Shanghai Jiao Tong University, Shanghai, 200030 China

**Keywords:** Premature ovarian insufficiency, Human amniotic epithelial cells, Single-nucleus RNA sequencing, Granulosa cells, IGF1R signaling

## Abstract

**Background:**

Premature ovarian insufficiency (POI) is defined by ovarian dysfunction and consequent decreased fertility. While follicular depletion is an acknowledged factor, dynamic changes in follicular subpopulations and single-cell-level niche remodeling remains largely unexplored. Human amniotic epithelial cells (hAECs) represent a promising regenerative approach for restoring ovarian function; however, the underlying mechanism in promoting follicular development remains unclear.

**Methods:**

We established a chemotherapy-induced POI mouse model and conducted single-nucleus RNA sequencing (snRNA-seq) to systematically characterize ovarian cellular heterogeneity, residual follicular development, and ovarian microenvironmental alteration. A human-derived cell projection model was established to track the distribution of transplanted hAECs. Furthermore, the functional role of IGF1R signaling in granulosa cells was validated using both in vitro culture assays and in vivo interventions.

**Results:**

We identified eight distinct ovarian cell types and uncovered stage-specific injury patterns: acute chemotherapy exposure induced massive loss of granulosa cells and oocytes, while the chronic phase was characterized by fibrotic accumulation and immune infiltration. Furthermore, residual follicles exhibited aberrant development trajectories and compromised progression, primarily due to disrupted granulosa cells –oocytes communication. Notably, hAEC transplantation significantly enhanced follicle survival, partially attenuated fibrosis and promoted ovarian structural restoration. Mechanistically, hAECs-derived IGFBP2 regulated IGF1R expression in granulosa cells, thereby reactivating the Akt/FoxO3A signaling pathway and downregulating the senescence marker P21.

**Conclusions:**

This study presents a time-resolved snRNA-seq atlas capturing both acute and chronic injury phases in a chemotherapy-induced POI model, combined with a human-mouse projection approach to track transplanted hAECs in damaged ovaries. Our study establishes a novel cell-based therapeutic strategy for partial ovarian functional recovery, in which hAEC-derived IGFBP2 restores granulosa cell function and intercellular communication by regulating the IGF1R/Akt/mTOR signaling axis.

**Supplementary Information:**

The online version contains supplementary material available at 10.1186/s13287-026-05064-8.

## Background

Premature ovarian insufficiency (POI) is defined as the loss of ovarian function before the age of 40, characterized by menstrual disturbance (oligomenorrhea or amenorrhea), elevated gonadotropin level, and estradiol deficiency. This condition leads to infertility and symptoms of estrogen deficiency, profoundly affecting the health and quality of life of young women [1]. Recent epidemiological study indicates the global prevalence of POI to be approximately 3.5%, with iatrogenic factors accounting for nearly 11.2% of cases [2]. Although chemotherapeutic drugs are crucial in oncology, they have serious side effects on reproductive system function [3]. A research reports that the risk of POI among young women undergoing receiving treatment is as high as 40%–70% [4].

Studies have indicated that the key mechanism underlying chemotherapy-induced POI is the accelerated depletion of the primordial follicle pool [5]. However, a single-oocyte transcriptomic analyses reveal that genotoxic agents primarily trigger oocyte apoptosis rather than activating follicular recruitment [6]. Additionally, chemotherapeutic agents inflict collateral damage on ovarian vasculature and stroma, fostering a pro-inflammatory microenvironment that further compromises follicular survival [7]. Despite these insights, the full spectrum of cellular heterogeneity and the bidirectional signals between oocytes and somatic cells following chemotherapy remains poorly defined.

Current management of POI relies heavily on hormone replacement therapy (HRT) to alleviate hypoestrogenic symptoms and reduce long-term sequelae such as osteoporosis and cardiovascular disease. However, HRT does not restore ovarian function and may increase cancer risk with prolonged use [8]. Stem cell-based therapies represent a promising alternative for restoring ovarian function. Human amniotic epithelial cells (hAECs) have garnered interest due to their non-tumorigenic nature, low immunogenicity, ethical acceptability, and ease of isolation [9]. Our previous studies have demonstrated that hAEC transplantation could restore ovarian function in murine models of chemotherapy-induced POI [10] through multiple mechanisms, including differentiation into granulosa cell [11], paracrine pathway [12], exosomal miRNA transfer [13], anti-inflammatory effects [14], and angiogenic promotion [15]. Importantly, preliminary clinical data also suggest the safety and efficacy of hAEC transplantation in partially restoring ovarian function [16]; yet, their precise impact on ovarian cellular function remains unclear.

The ovary is a complex, dynamic ecosystem where folliculogenesis relies on the crosstalk between germ and somatic cells, which is essential for oocyte maturation and steroidogenesis [17]. Chemotherapy agents disrupt these coordinated interactions, but the molecular details are obscured by the diversity of ovarian cells. Single-cell RNA sequencing (scRNA-seq) and single-nucleus RNA sequencing (snRNA-seq) are powerful tools for resolving tissue heterogeneity, uncovering cell-type-specific responses and cell-cell signaling networks in physiological and pathological states. Currently, scRNA-seq has been used to study human ovarian remodeling [18] and developmental defects [19], and snRNA-seq has revealed mechanisms of cigarette smoke-induced diminished ovarian reserve in mice [20].

In this study, we preformed snRNA-seq to construct a high-resolution cellular atlas of the ovary in a murine model of chemotherapy-induced POI. Through longitudinal analysis, we captured the spatiotemporal evolution of ovarian damage and repair, identifying impaired follicular maturation and disrupted intercellular communication as hallmarks of chemotherapy-induced POI. Furthermore, we established a novel method based on snRNA-seq data to track the homing of transplanted hAECs and identified the IGF1R/Akt/mTOR pathway as a core mechanism of hAECs-mediated ovarian functional recovery.

## Methods

### Animal experiments

A total of 88 female C57BL/6J mice (aged 6–8 weeks) were purchased from Shanghai Lingchang Biotechnology Co., Ltd. (Shanghai, China). All mice were housed under a 12-hour light/dark cycle at 22 ± 1 °C with ad libitum access to food and water. Following anesthesia induction with tribromoethanol (20 µL/g, Beran, China) administered intraperitoneally, blood and ovarian samples were collected. Subsequently, all mice were euthanized by cervical dislocation. Every effort was made to alleviate animal suffering and reduce the number of animals used. All animal experiments adhere to the ARRIVE guidelines 2.0.

### Culture and identification of hAECs

Term placentas with intact fetal membranes were obtained from healthy donors following informed consent and negative screening for hepatitis B, hepatitis C, and HIV. The amniotic membrane was manually separated from the chorion, cut into small segments, and rinsed thoroughly with phosphate-buffered saline (PBS). Tissue segments were digested using 0.25% trypsin-EDTA (Gibco, Grand Island, NY, USA) for 25 min at 37 °C. The digested suspension was filtered through a 40 μm cell strainer and centrifuged at 300 × g for 5 min at room temperature. The resulting cell pellet was resuspended and seeded into 100 mm culture dishes containing DMEM/F12 medium (Gibco) supplemented with 10% fetal bovine serum (FBS, Gibco), 2 mM glutamine, 100 µg/mL streptomycin, 100 U/mL penicillin, and 10 ng/mL recombinant human epidermal growth factor (EGF, ProSpec). Cells were cultured at 37 °C in a humidified incubator with 5% CO₂.

hAECs were characterized using flow cytometry and immunofluorescence. For flow cytometric analysis, cells were harvested, washed, and stained with the following fluorescently conjugated primary antibodies: SSEA4 (pluripotent marker, 1:1000, BioLegend, San Diego, CA, USA), CD324 (epithelial marker, 1:1000, BioLegend), CD146 (mesenchymal marker, 1:1000, BioLegend), and HLA-DR (1:1000, BioLegend) for 30 min at 4 °C in the dark. After staining, cells were washed with cold PBS and analyzed immediately using a FC500 flow cytometer (Beckman Coulter). For immunofluorescence staining, cells were fixed with 4% paraformaldehyde (PFA) for 15 min at room temperature. After per-meabilization with 0.1% Triton X-100 (Sigma-Aldrich, St. Louis, MO, USA), cell were incubated with the following primary antibodies at 4 °C for overnight: OCT4 (stem cell maker, 1:200, Boster Biological Technology), CK18 (epithelial marker, 1:200, Boster), CK7 (epithelial marker, 1:200, Boster), N-cadherin (mesenchymal marker, 1:200, Boster) and Vimentin (mesenchymal marker, 1:200, Boster). Cells were then incubated with Alexa Fluor 488 and 594-conjugated secondary antibody (1:1000, Thermo Fisher Scientific) for 1 h at room temperature. Nuclei were counterstained with DAPI (1:1000, Sigma-Aldrich), and fluorescence signals were visualized using a confocal microscope (Leica, Wetzlar, Germany). These results showed that the hAECs were positive for stem cell markers (SSEA4, OCT4) and epithelial markers (CD324, CK18, CK7), while being negative for mesenchymal markers (CD146, N-cadherin, Vimentin), as shown in the Supplementary Fig. 1.

### Animal grouping and tissue collection

A total of 28 female mice (18–20 g) were randomly divided into two groups: a sham control group (Sham, *n* = 7) and a chemotherapy-induced POI group (Cy, *n* = 21). Mice in the POI group received a single intraperitoneal injection of busulfan (30 mg/kg; Sigma-Aldrich, St. Louis, MO, USA) and cyclophosphamide (120 mg/kg; Hengrui, Jiangsu, China). The sham controls were injected with an equal volume of PBS.

Among the POI mice, three subgroups were established based on subsequent treatments. In the hAEC transplantation group (Cy-hAECs, *n* = 7), mice received a tail vein injection of 2 × 10⁶ hAECs suspended in 150 µL PBS on day 7 post-chemotherapy. Mice in the chemotherapy injury-21 Day group (Cy-21 Day, *n* = 7) received a tail vein injection of 150 µL PBS on day 7 post-chemotherapy. The other chemotherapy-treated mice were designated as the chemotherapy injury-3 Day group (Cy-3 Day, *n* = 7).

Ovarian tissues were harvested at the following time points: from the Sham group, and at 3 days and 21 days post-chemotherapy, as well as 14 days post-transplantation. For morphological evaluation, ovarian tissues were fixed, paraffin-embedded, sectioned, and used for follicle counting and fibrosis assessment (*n* = 4 per group). For snRNA-seq, ovarian tissues were processed for nuclear isolation (*n* = 3 per group). Each replicate per group was generated by pooling both ovaries from an individual mouse, which was then processed as a single sample.

### Dissociation of ovaries

For snRNA-seq, the ovarian tissues from mice were dissociated using the Multi Tissue Dissociation Kit 1 (Miltenyi Biotec Inc., USA). For each ovary, an enzyme mixture was prepared in a gentleMACS C Tube (Miltenyi Biotec Inc., USA) by combining 2.35 mL of serum-free DMEM (Biosharp, China), 100 µL of Enzyme D, 50 µL of Enzyme R, and 12.5 µL of Enzyme A, according to the manufacturer’s protocol. The ovarian tissue was placed into the tube containing the enzyme mixture, and the tube was securely closed. The C Tube was then inverted and mounted onto the sleeve of the gentleMACS Octo Dissociator with Heaters (Miltenyi Biotec Inc., USA), and the program 37C_Multi_A was executed. Dissociation efficiency and cell status were assessed by cell counting.

### Isolation of single cell nuclei

Single-nucleus isolation from dissociated ovaries was performed following a modified 10×Genomics nuclei isolation protocol, with all steps carried out on ice. Each ovarian tissue sample was placed in a wide‐bore pipette tip (Rainin, USA) containing 1 mL of ice‐cold lysis buffer (10 mM Tris–HCl, pH 7.4, 10 mM NaCl, 3 mM MgCl₂, 0.1% Nonidet™ P40 Substitute (Sigma‐Aldrich, USA) in nuclease‐free water). Then, 1 mL of Nuclei Wash and Resuspension Buffer (1×PBS with 1.0% BSA and 0.2 U/mL Protector RNase Inhibitor (Sigma‐Aldrich, USA)) was added using a regular‐bore pipette tip and mixed gently by pipetting. The nuclei were pelleted and resuspended in an appropriate volume of the same buffer to achieve a target concentration of 1000 nuclei/mL. After gentle pipetting to ensure complete resuspension, an aliquot of nuclei was diluted 1:1 with Trypan Blue, and the nuclei concentration was determined using a Countess II FL Automated Cell Counter (Thermo Fisher Scientific, USA).

### Preparation of single-cell nuclei cDNA library and sequencing

The number and status of nuclei were determined by staining with AO/PI staining. Subsequently, the nuclei were processed through the 10×Genomics Platform using the Chromium Next GEM Single Cell 3ʹ Reagent Kits. The nuclei were loaded onto 3ʹ library chips for the Chromium Next GEM Single Cell 3ʹ Library Kit, following the protocol. Briefly, the Next Gem technology was adopted by 10× to partition the single cell suspension into Gel Beads in Emulsion in the 10×Chromium controller instrument, under the process of cell lysis and barcoded reverse transcription of RNA. Amplification, shearing, 5ʹ adaptor, and sample index PCR were subsequently performed. Approximately 16,000 cells were loaded onto the nuclei. The libraries were sequenced on an Illumina NovaSeq 6000.

### Processing and quality control of snRNA-seq data

SnRNA-seq data were demultiplexed and converted to FASTQ format using Illumina bcl2fastq software. Sample demultiplexing, barcode processing, and single-cell 3ʹ gene counting were performed using the Cell Ranger Single-Cell Software Suite (version 5.0). The cDNA inserts were aligned to the mm10/GRCm38 reference genome. Only reads that were confidently mapped, non-PCR duplicates, and contained valid barcodes and unique molecular identifiers were retained for constructing the gene-barcode matrix. Subsequent analyses, including quality control filtering, highly variable gene identification, dimensionality reduction, unsupervised clustering, and differential gene expression analysis, were analyzed by R software (version 3.6.1) with the Seurat package (version 3.2.0). The median and average sequencing depth per nucleus were 2338 and 2,342 unique molecular identifiers (UMIs), respectively. To exclude low-quality nuclei and those potentially affected by dissociation stress, we applied a filter based on mitochondrial UMI content (> 20%) and expression of hemoglobin (Hbb) family genes (> 10%). We mitigated ambient RNA contamination by excluding nuclei with high expression of ribosomal protein genes during the identification of highly variable genes. After filtering, the expression matrix was normalized by total cellular expression, scaled by a factor of 10,000, and log-transformed. In addition to standard quality control filtering based on mitochondrial gene percentage and unique gene counts, we employed a formal doublet-detection tool DoubletFinder (version 2.0), to identify and remove putative doublets from the single-cell RNA sequencing data. Briefly, we followed the official standard workflow to complete doublet removal.

### Cell clustering, doublet calling, and annotation

Dimensionality reduction and unsupervised clustering were conducted using the standard Seurat workflow. The SCTransform function was applied to normalize the single-cell gene expression data and identify highly variable genes (HVGs). Mitochondrial genes, dissociation-induced genes, and HLA genes were excluded from the HVG set for downstream analysis. The influence of mitochondrial transcript proportion was regressed out using SCTransform with the parameter vars.to.regress = “percent.mt”.

A principal component analysis (PCA) was performed using the RunPCA function with default parameters to reduce noise and capture major sources of variation. To mitigate batch effects across samples, Harmony (version 1.0) was applied to the PCA embedding using default settings. Uniform Manifold Approximation and Projection (UMAP) and graph-based clustering were subsequently performed in the harmonized space to visualize and identify cell clusters. Cell types were annotated based on differentially expressed genes (DEGs) and well-established marker genes reported in the literature.

### Cell–cell interaction analysis

Cell–cell interactions were analyzed using CellPhoneDB (version 3.0), with analysis restricted to protein–protein interaction subsets. The thickness of each connecting line indicates the number of potentially activated ligand–receptor pairs between the corresponding cell types.

### Establishment of projection model of hAECs

We constructed an integrated reference atlas of mouse ovarian cell states from PBS- and hAEC-treated samples. To project the transplanted human cells into this reference UMAP space, we employed a CCA-based algorithm. A critical step was separate read alignment: human-mapped reads were assigned to the human transcriptome, and mouse-mapped reads to the mouse transcriptome, ensuring accurate species assignment. Projection confidence was assessed using per-cell correlation scores. Furthermore, the stringent pre-processing filters applied to the entire dataset served as essential controls to minimize the impact of doublets and ambient RNA on the results. This approach enabled us to distinguish and visualize the transplanted human cells within the host ovarian niche.

This study utilizes a preprocessed single-cell transcriptomic dataset that has undergone dimensionality reduction and clustering as a reference, performing cell projection analysis based on the Seurat platform. First, highly variable genes (HVGs) shared between the reference dataset and the new dataset (query) are extracted to ensure consistency in the gene set. Subsequently, the FindIntegrationAnchors function is employed to identify cell anchors between the reference and query datasets, with the parameter k.filter = 50 set to retain only the top 50 highest-confidence anchors for downstream analysis. Based on the filtered anchors, the IntegrateData function is used to integrate the query dataset and project it into the UMAP space of the reference atlas, while preserving the original UMAP coordinates of the reference unchanged. Ultimately, this approach enables the visualization and localization of the query cells within the existing low-dimensional space.

### Transmission electron microscopy

Fresh ovarian tissues were collected from Sham group and Cy-21 Day group, and promptly dissected into small fragments (approximately 1 mm³), which were immediately fixed in a solution of 2.5% glutaraldehyde in 0.1 M cacodylate buffer at room temperature. The fragments were then washed three times in 0.1 M cacodylate buffer, each wash lasting 15 min. Following this, the samples were post-fixed with 1% osmium tetroxide for 1.5 h at 4 °C, and subsequently rinsed three times in ddH₂O for 5 min each.

After dehydration, the samples were embedded in pure EPON resin and polymerized sequentially at 35 °C for 24 h, 45 °C for 24 h, and finally 60 °C for 24 h to form solid blocks. Ultrathin sections were prepared, mounted on copper grids, and stained with uranyl acetate and lead citrate for observation of mitochondrial ultrastructure using transmission electron microscopy (TEM).

### Ovarian histological analysis

Ovarian tissues were fixed in 4% PFA, dehydrated through a graded ethanol series, cleared in xylene, and embedded in paraffin. Serial sections were cut at a thickness of 5 μm for subsequent staining.

Hematoxylin and eosin (HE) staining was performed to evaluate follicular development. Follicle counting was conducted under light microscopy by two independent researchers in a blinded manner, examining every fifth section throughout the ovary. Follicles were classified based on established morphological criteria. A primordial follicle refers to a single fusiform oocyte surrounded with GCs. A primary follicle indicates the unit of an oocyte surrounded by at least three cubic-shape GCs. A secondary follicle is characterized by an oocyte surrounded by at least two layers of GCs with follicular cavity deficiency. Mature follicles (also called antral follicles) contain at least two layers of GCs with an evident follicular cavity.

To assess ovarian fibrosis, Sirius red and Masson’s trichrome staining were employed. For Sirius red staining, deparaffinized and rehydrated sections were stained with Picric acid–Sirius red (PSR) solution (0.1% w/v Sirius Red F3BA, Sigma-Aldrich in saturated picric acid) for 40 min at room temperature. Sections were then treated with 0.5% glacial acetic acid and 0.05 M hydrochloric acid, followed by four washes. After careful removal of residual acid, sections were rapidly dehydrated in 100% ethanol. All PSR staining procedures were consistent in duration across experiments to minimize variation in staining intensity. Masson’s trichrome staining was performed according to the manufacturer’s instructions (Yeasen, Shanghai, China) to evaluate collagen deposition in ovarian tissues.

### Immunofluorescence staining

Following deparaffinization, antigen retrieval was performed by heating ovarian sections at 95 °C in EDTA buffer. The sections were then sequentially blocked with 5% bovine serum albumin (BSA) and permeabilized with 0.3% Triton X-100. After washing with PBS, the sections were incubated overnight at 4 °C with the following primary antibodies: anti-IGF1R/SP1/ZP2 (dilution 1:250-1:100, Proteintech 20254-1/21962-1/21832-1), IGF1R/AMH/P21 (dilution 1:250, Proteintech 20254-1/14461/CST 2947 T), and IGF1R/Nr2f2/α-SMA (dilution 1:250, Proteintech 20254-1/Abcam ab211777/CST 19245 T). After three washes with PBS, the sections were incubated for 60 min at room temperature in the dark with the following secondary antibodies diluted in 2% BSA: Alexa Fluor^®^ 488 goat anti-rabbit IgG (1:2000), Alexa Fluor^®^ 647 goat anti-rabbit IgG (1:2000), Cy3-conjugated goat anti-rabbit IgG (1:200). Finally, the sections were washed again with PBS and mounted with antifade mounting medium containing DAPI for nuclear counterstaining.

### Collection and analysis of hAEC conditioned medium

The conditioned medium of human amniotic epithelial cells (hAEC-CM) was collected and subjected to cytokine array analysis. Briefly, 2 × 10⁶ hAECs were seeded into a 100 mm culture dish. After 24 h, the complete culture medium was replaced with 10 mL of serum-free DMEM/F12 medium. Following another 24 h of culture, the supernatant was gently collected, filtered through a 0.22 μm membrane, and concentrated using Amicon Ultra-15 centrifugal filters with a 3 kDa molecular weight cutoff. The hAEC-CM was concentrated 20-fold relative to the original volume. A control medium was prepared identically using DMEM/F12 without cells.

A cytokine antibody array (GSHCAA-440, RayBiotech, Norcross, GA, USA) was used to assess the expression of IGF-related cytokines in the concentrated hAEC-CM, according to the manufacturer’s instructions.

Western blot was performed to detect IGFBP2 (1:1000, Proteintech 11065-3), IGFBP3 (1:1000, Proteintech 10189-2), and IGFBP6 (1:1000, Proteintech 67567-1) expression in concentrated hAEC-CM samples obtained from four different donors. Basic DMEM/F12 medium, processed similarly but without cells, served as the negative control.

### Fluorescence tracing of transplanted stem cells

A lentiviral vector encoding EGFP (GL194, pSLenti-SFH-EGFP-P2A-Puro-CMV-MCS-30×FLAG-WPRE; Obio Technology, Shanghai, China) was used to transduce hAECs. Transfection efficiency exceeded 90%, as confirmed by fluorescence microscopy (Leica, Wetzlar, Germany). To enrich GFP-positive cells, puromycin (5 µg/mL; Yeasen) was applied for 72 h.

To establish the chemotherapy-induced POI model, 10 female mice (18–20 g) received a single intraperitoneal injection of busulfan (30 mg/kg) and cyclophosphamide (120 mg/kg). Subsequently, the mice were randomly divided into two groups: hAECs treatment group (Cy‑hAECs + GFP, *n* = 5) received 2 × 10⁶ GFP-labeled hAECs resuspended in 150 µL PBS via tail vein injection, while the control group (Cy, *n* = 5) received an equal volume of PBS alone.

To quantify cell retention, ovaries were harvested 14 days after transplantation and processed into single-cell suspensions. After erythrocyte lysis, nonspecific binding was blocked using Human TruStain FcX™ (BioLegend) for 5 min at room temperature. GFP‑positive cells were detected by flow cytometry. Concurrently, frozen ovarian sections were prepared to visualize the spatial distribution of GFP fluorescence.

### In vitro experiments

KGN cells were maintained in DMEM/F12 medium containing 10% FBS, 100 U/mL penicillin, and 100 µg/mL streptomycin at 37 °C in a 5% CO₂ atmosphere. To induce cellular senescence, cells were cultured in serum free medium for 24 h. Subsequently, the experimental groups were incubated with hAEC-CM, hAEC-CM containing an IGFBP2 antibody, and IGF1 supplementary, while the control group received no further treatment. After another 24 h, the cells were harvested for subsequent analyses.

### Western blot analysis

Proteins were extracted from cells using RIPA lysis buffer (Beyotime, Shanghai, China), and protein concentrations were determined with a BCA protein assay kit (Boster, Wuhan, China). Equal amounts of protein were separated by SDS-PAGE and transferred onto PVDF membranes (Millipore, USA). The membranes were blocked with 5% non-fat milk in TBST for 1 h at room temperature and then incubated overnight at 4 °C with the following primary antibodies: anti-IGF1R (1:500, Proteintech 20254-1), anti-SP1 (1:500, Proteintech 21962-1), anti-P21 (1:1000, Proteintech 82669-2), anti-p-Akt/Akt (1:1000, CST 9275 S/4691S), anti-p-mTOR/mTOR (1:1000, CST 5536 S/2983S), anti-p-FoxO3A/FoxO3A (1:500, SAB 12199/40937) and anti-GAPDH/Tubulin (1:10,000, Yeasen).

After washing, the membranes were incubated with horseradish peroxidase-conjugated secondary antibodies (dilution 1:1000) for 1 h at room temperature. Protein bands were visualized using an enhanced chemiluminescence kit (Santa Cruz Biotechnology, USA), and band intensities were quantified with ImageJ software.

### Enzyme-linked immunosorbent assay (ELISA)

Serum and ovarian tissue lysates were collected from mice in different treatment groups to determine IGF1 protein levels in both the peripheral circulation and ovarian tissue. Serum concentrations of estradiol (E2), follicle-stimulating hormone (FSH), and anti-Müllerian hormone (AMH) were quantified using commercial ELISA kits according to the manufacturers’ protocols.

### Ovarian function and fertility assessment

A total of 50 female mice were randomly assigned to a sham group and a chemotherapy-induced POI group. Mice in the chemotherapy group (Cy, *n* = 40) received a single intraperitoneal injection of busulfan (30 mg/kg) and cyclophosphamide (CTX, 120 mg/kg). The sham group (*n* = 10) received an equal volume of PBS. The POI mice were further randomized into four treatment groups: Cy-PBS group (*n* = 10), received PBS only; Cy-hAEC group (*n* = 10), administered 2 × 10⁶ hAECs in 100 µL PBS via tail vein injection; Cy-PPP group (*n* = 10), treated with picropodophyllin (PPP, 15 mg/kg/day) via intraperitoneal injection for 14 days; Cy-IGF1 group (*n* = 10), treated with IGF1 (20 µg/kg/day) via intraperitoneal injection for 14 days.

Estrous cycles were monitored by vaginal cytology. Vaginal smears were collected daily for 7 consecutive days each morning. A pipette was used to gently flush the vagina with sterile saline warmed to 40 °C. Smears were mounted on glass slides, stained with Giemsa solution, and examined under a microscope. Estrous cycle staging was performed independently by two investigators using established criteria.

Ovarian follicle development was evaluated using HE staining. Ovaries from each group were fixed in 4% PFA, embedded in paraffin, sectioned, and stained with HE for histological quantification of follicles (*n* = 5 per group).

To assess fertility, female mice from each group were mated with proven fertile male C57BL/6 mice (*n* = 5 per group). Fertility was assessed by examining embryonic development at 16.5–17.5 days after a vaginal plug was detected. Pregnant mice were anesthetized via an intraperitoneal injection of tribromoethanol, and the number and morphology of embryos in their uteri were examined.

### Statistical analysis

Image acquisition and histological analysis were performed in a blinded manner with respect to treatment groups. Data are presented as mean ± standard error of the mean (SEM). Comparisons between two groups were made using Student’s t-test; multiple group comparisons were conducted using one-way analysis of variance (ANOVA) followed by Bonferroni correction. Pregnancy rates were compared using Fisher’s exact test. All analyses were performed with GraphPad Prism version 9 (GraphPad Software, San Diego, CA, USA). A p-value of less than 0.05 was considered statistically significant.

## Results

### snRNA-seq uncovers temporal dynamics of ovarian cell populations after chemotherapy

**Fig. 1:**
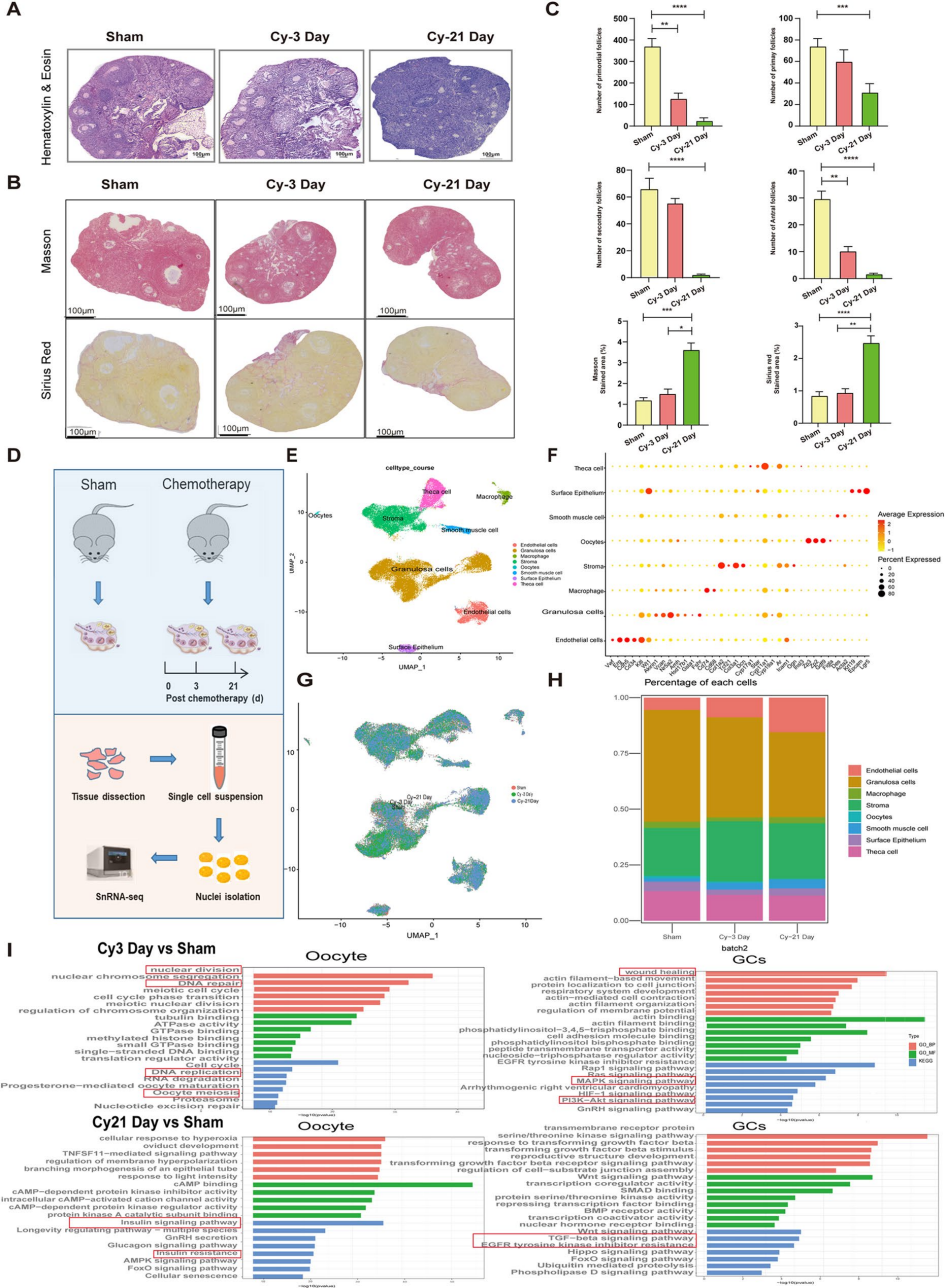
Single-nucleus RNA sequencing reveals the impact of chemotherapy on ovarian cell composition and function. **A** Representative HE-stained ovarian sections from mice in different treatment groups. **B** Masson’s trichrome and Sirius red staining revealing collagen deposition in ovarian tissues. **C** Quantification of follicles at each developmental stage and extent of fibrosis across groups (*n* = 4 mice per group). **D** Schematic diagram of the snRNA-seq experimental design. Ovaries were collected from three groups (*n* = 3 mice per group). **E** UMAP visualization of all ovarian cells, with eight distinct clusters identified based on signature gene expression and distinguished by colors. Each dot represents an individual cell. **F** Annotation of major cell clusters using established marker genes. **G** UMAP plots showing cell distributions across experimental groups, indicating no major differences in overall cellular architecture. **H** Proportions of ovarian cell clusters in each group. **I** GO and KEGG enrichment analyses of differentially expressed genes in oocytes and granulosa cells at different time points after chemotherapy. Data are presented as mean ± SEM; *P < 0.05, **P < 0.01, ***P < 0.001, ****P < 0.0001. Scale bars: 100 μm

Consistent with established model of chemotherapy-induced ovarian damage, the morphological analysis at Days 3 and 21 post-chemotherapy confirmed progressive follicular depletion and fibrotic restructuring (Fig. 1A–C). To elucidate cell-type-specific alterations, we conducted snRNA-seq on nuclear suspensions from Sham, Cy-3 Day, and Cy-21 Day groups (Fig. 1D). After quality control, 70,520 high-quality nuclear transcriptomes were analyzed, with median detection of 1,774 genes per nucleus (Supplementary File 1). Graph-based clustering and UMAP visualization identified eight major cell clusters, annotated using lineage-specific markers, including Endothelial cells (vWF), Granulosa cells (Nr5a2), Macrophage (Cd74), Stroma (Col1a2), Oocyte (ZP3), Smooth muscle cells (Des), Surface Epithelium cells (Lgr5), and Theca cells (Cyp11a1), as shown in Fig. 1E-F. Comparative cell composition analysis revealed a significant depletion of oocytes and granulosa cells during the acute injury phase (Day 3 post-chemotherapy), concurrent with an expansion of stromal and smooth muscle cell populations in the subsequent ovarian function remodeling phase (Day 21 post-chemotherapy) (Fig. 1G-H).

Gene Ontology and KEGG analyses revealed time-dependent functional shifts. DEGs in oocytes at Day 3 were enriched in chromatin segregation, DNA repair, and replication processes. In granulosa cells, acute injury was linked to MAPK and PI3K/Akt pathway activation, whereas DEGs predominantly enriched for metabolic regulators and the TGF-β signaling pathway at Day 21 (Fig. 1I). Collectively, these stage-specific transcriptional changes demonstrate that chemotherapy causes extensive and functional impairment to ovarian follicular cells.

### Chemotherapy disrupts the developmental trajectory of granulosa cells and POI-associated gene expression in a single-cell atlas

**Fig. 2:**
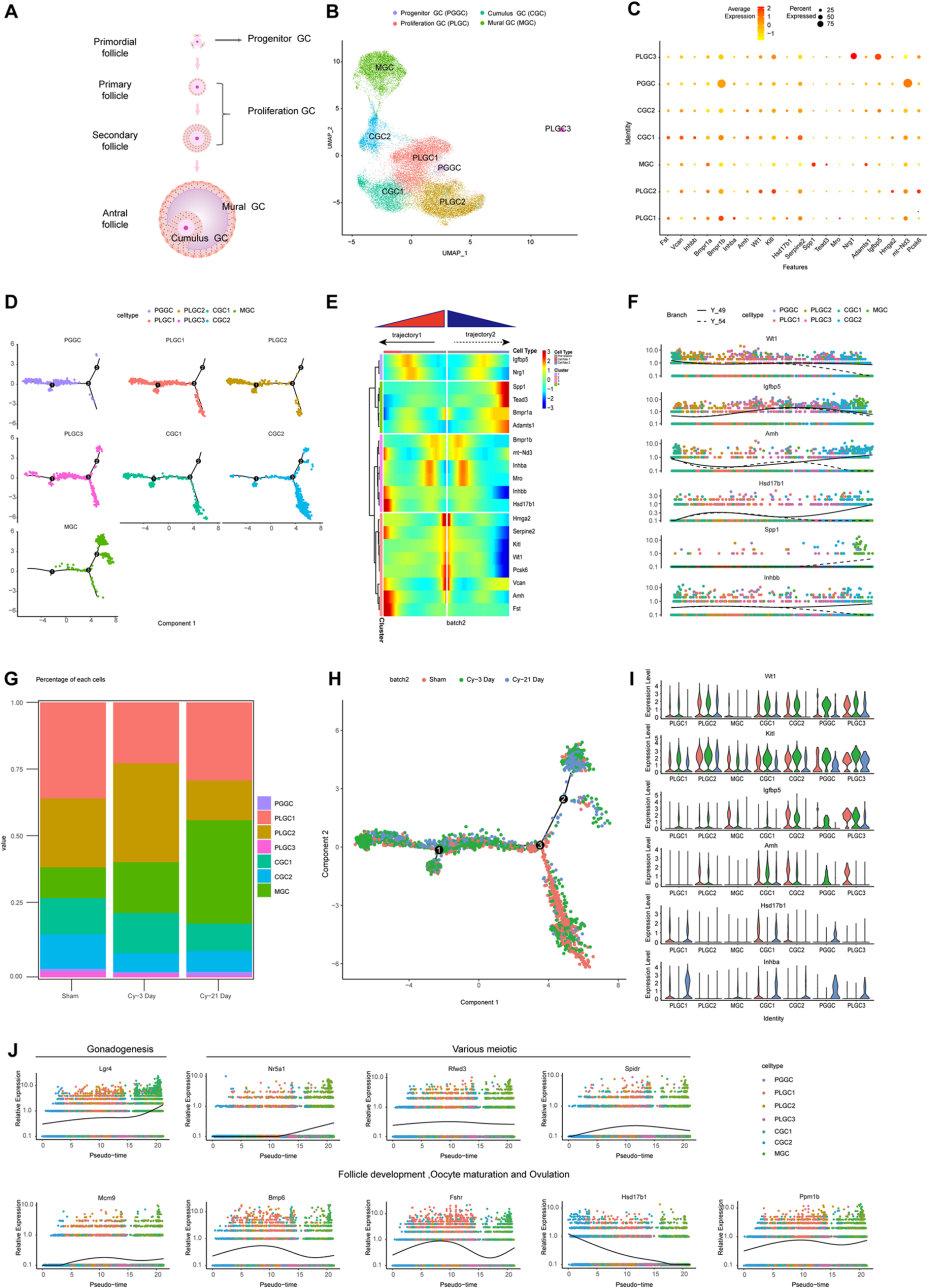
Heterogeneity and developmental trajectory of granulosa cells (GCs). **A** Schematic representation of granulosa cells at different follicular developmental stages. **B** UMAP visualization of granulosa cell subtypes, including primordial GCs (PGGCs), proliferating GCs (PLGCs), cumulus GCs (CGCs), and mural GCs (MGCs). Colors represent distinct cell types. **C** Dot plot showing the average expression levels and percentage of cells expressing representative marker genes in each GC subtype. **D** Pseudotemporal trajectory of GCs reconstructed using Monocle2. Cells are colored by cluster identity. Diffusion mapping reveals conserved progression across subtypes. **E** Heatmap of state-specific marker genes identified along the pseudotime trajectory, showing expression dynamics from the root toward Terminal 1 (mural GCs) and Terminal 2 (cumulus GCs). **F** Expression kinetics of key GC genes along two pseudotime trajectories: Trajectory 1 (dotted line) and Trajectory 2 (solid line). Points represent individual cells colored by subtype. **G** Stacked bar plot illustrating the compositional distribution of GC clusters across developmental stages and treatment groups. **H** Pseudotime analysis highlights GC heterogeneity across developmental stages following chemotherapy exposure. **I** Violin plots showing expression levels of key genes in GCs at different chemotherapy time points. **J** Pseudotemporal expression patterns of POI-related pathogenic genes in granulosa cells

To investigate chemotherapy-induced follicular damage, we analyzed granulosa cells (GC) subpopulations based on the marker genes established by Fan et al.^18^. GCs were classified into four subtypes, including progenitor GCs (PGGC; high Wt1/Egr4, low Vcan/Fst), proliferating GCs (PLGC; high Amh/Ki-67/Wt1), cumulus GCs (CGC; high Vcan/Fst/Igfbp2/Htra1/Inhbb/Ihhba) and mural GCs (MGC; low Wt1/Egr4, high Krt18/Cited2/Liph/Akirin1). Using these markers, the seven GC clusters identified in this study were annotated as PGGC, proliferating GCs (PLGC1, PLGC2, PLGC3), MGC, and cumulus GCs (CGC1, CGC2) (Fig. 2A–C).

Pseudotemporal trajectory analysis using Monocle revealed two differentiation branches within the GC lineage (Fig. 2D). PGGC maintenance was governed by Hmga2, Kitl, Wt1, and Vcan. Differentiation into PLGC was driven by Igfbp5 and Nrg1, followed by bifurcation into CGCs (expressing Hsd17b1/AMH/Fst) or MGCs (expressing Spp1/Tead3/Adamts1), which support oocyte development and steroidogenesis, respectively (Fig. 2E–F).

To assess the impact of chemotherapy on the developmental trajectory granulosa cells, we performed comparative analysis across experimental groups. At Day 3 post-chemotherapy, PGGCs were significantly reduced, whereas PLGCs (Kitl-high) expanded. By Day 21, PGGCs remained low, MGCs increased and CGCs decreased (Fig. 2G–I). These results indicate that chemotherapy severely disrupts the development of GC, leading to abnormal differentiation trajectories.

We further mapped POI-related genes from clinical cohorts [21] onto GC developmental trajectories. Lgr4 was essential throughout GC maturation; its loss impaired general GC function. Bmp6 and Fshr dysregulation specifically disrupted PLGC formation, linked to early ovarian failure. Meanwhile, Hsd17b1 and Nr5a1 defects impaired both CGC and MGC differentiation, leading to defective steroidogenesis and hormonal imbalance (Fig. 2J). These findings enable precise stratification of POI subtypes and inform targeted therapeutic strategies based on developmental pathogenesis.

### Chemotherapy disrupts oocyte-granulosa cell crosstalk, characterized by insulin pathway activation

**Fig. 3:**
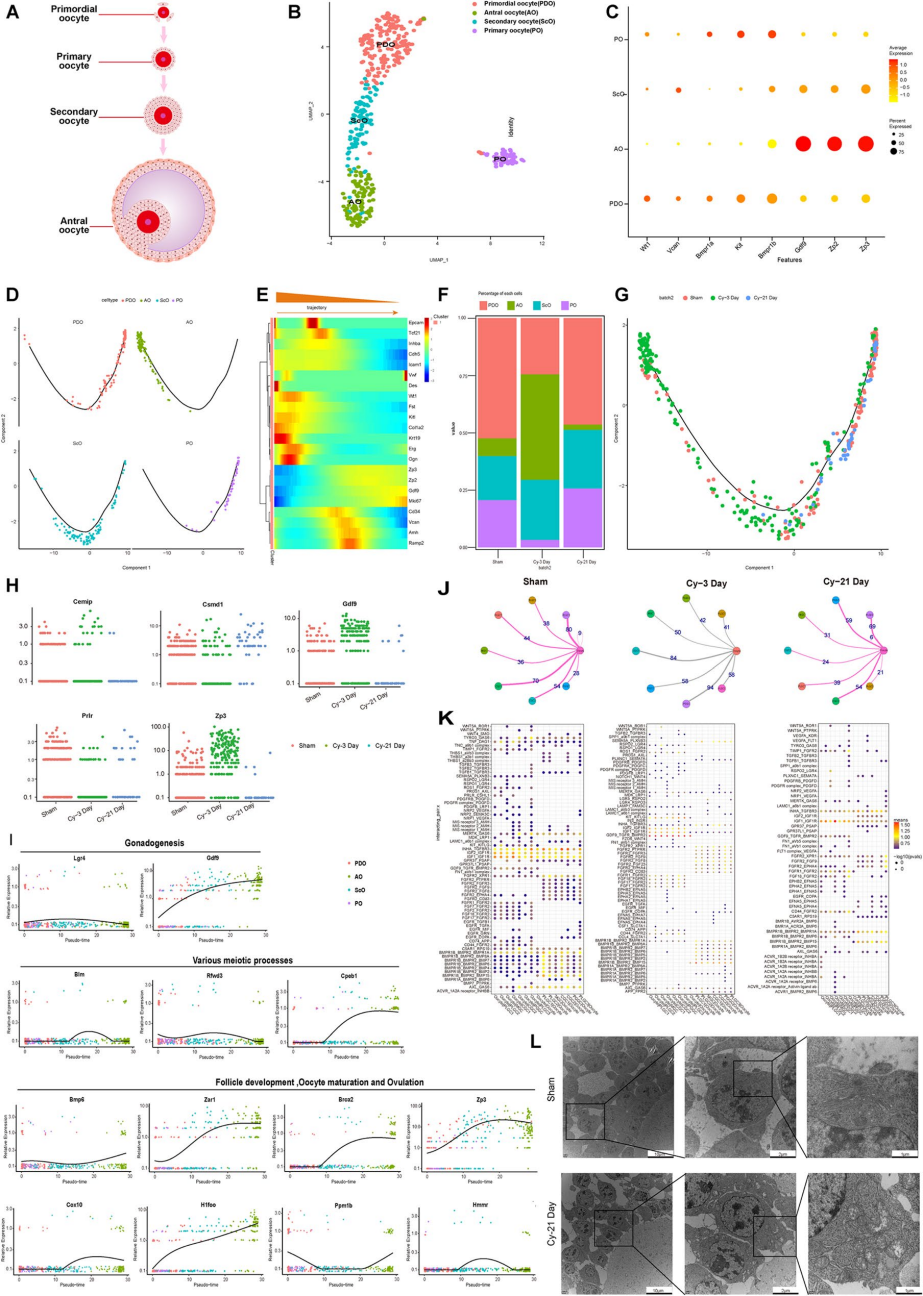
Heterogeneity and developmental trajectory of oocytes. **A** Schematic representation of oocytes at different follicular developmental stages. **B** UMAP visualization of four oocyte subpopulations, including primordial oocytes (PDO), primary oocytes (PO), secondary oocytes (ScO) and antral oocytes (AO). **C** Dot plot showing the expression and proportion of cells expressing established marker genes across oocyte developmental stages. **D** Pseudotemporal trajectory of oocyte subtypes inferred by Monocle 2 using Seurat-processed data, with cells colored by subtype identity. **E** Heatmap of genes with dynamic expression along pseudotime, grouped into four clusters corresponding to PDO, PO, ScO, and AO stages. **F** Stacked bar plot illustrating the distribution of oocyte subtypes across developmental stages and treatment groups. **G** Expression trends of signature oocyte genes along pseudotime, displayed as scatter plots. **H** Comparison of stage-specific gene expression patterns in oocytes across treatment groups. Each point represents a single cell. **I** Pseudotemporal expression patterns of POI-related pathogenic genes in oocytes. **J** Strength of cell-cell communication between granulosa cells and oocytes across the three experimental groups. **K** Dot plot depicting the mean expression level and percentage of cells expressing selected ligand-receptor pairs involved in granulosa cell-oocyte crosstalk, analyzed per sample group. **L** Transmission electron microscopy (TEM) images comparing ultrastructural features of oocytes and granulosa cells in antral follicles between Sham and Cy-21 Day groups. Scale bars: 1 μm, 2 μm, and 10 μm

To investigate chemotherapy-induced oocyte pathology, we reconstructed developmental trajectories using pseudotemporal ordering. Four distinct subpopulations were identified, including primordial oocyte (PDO), primary oocyte (PO), secondary oocyte (ScO), and antral oocyte (AO) (Fig. 3A–B). Stage-specific molecular signatures revealed that Wt1 and Vcan were highly expressed in PDOs, whereas Bmpr1a and Kit showed progressive upregulation during the PDO-to-PO transition. Bmpr1b and Gdf9 were increased in ScO, and ultimately the robust high expression of Zp2 and Zp3 in AO (Fig. 3C–E).

Subsequently, we evaluated chemotherapy-induced alterations in oocyte development by quantifying stage-specific population. At Day 3 post-chemotherapy, PDO and PO populations were markedly depleted, whereas ScO and AO proportions expanded (Fig. 3F–H). Projection of known POI-associated genes onto these trajectories revealed distinct enrichment patterns, Bmp6, Cox10 and H1foo in AOs; Zar1 and Hmmr in ScOs; and Ppm1b for PDO maintenance (Fig. 3I).

Cell-cell communication analysis demonstrated that chemotherapy initially enhanced overall oocyte-granulosa cell (OC-GC) signaling at Day 3 post-chemotherapy. However, oocyte-mediated regulation of granulosa cells was broadly suppressed, marked by significant reductions in Inha–Tgfbr3 and Bmp–Bmpr signaling, alongside pronounced upregulation of Kit–Kitlg and insulin (Igf1/Igf2–Igf1r) pathways. By Day 21, global OC-GC communication declined. Although Bmp-Bmpr signaling showed partial recovery, sustained Igf1-Igf1r activation suggested a central role for insulin signaling in chemotherapy-induced ovarian dysfunction (Fig. 3J-K). Transmission electron microscopy further revealed ultrastructural alterations in the mitochondria of granulosa cells, as well as abnormal junctions between granulosa cells and oocytes within antral follicles at Day 21 after chemotherapy (Fig. 3L). These findings suggest that chemotherapy disrupts follicular development by impairing oocyte maturation and disturbing granulosa cell–oocyte crosstalk.

### Evaluating the impact of hAEC transplantation on ovarian function using snRNA-seq

**Fig. 4:**
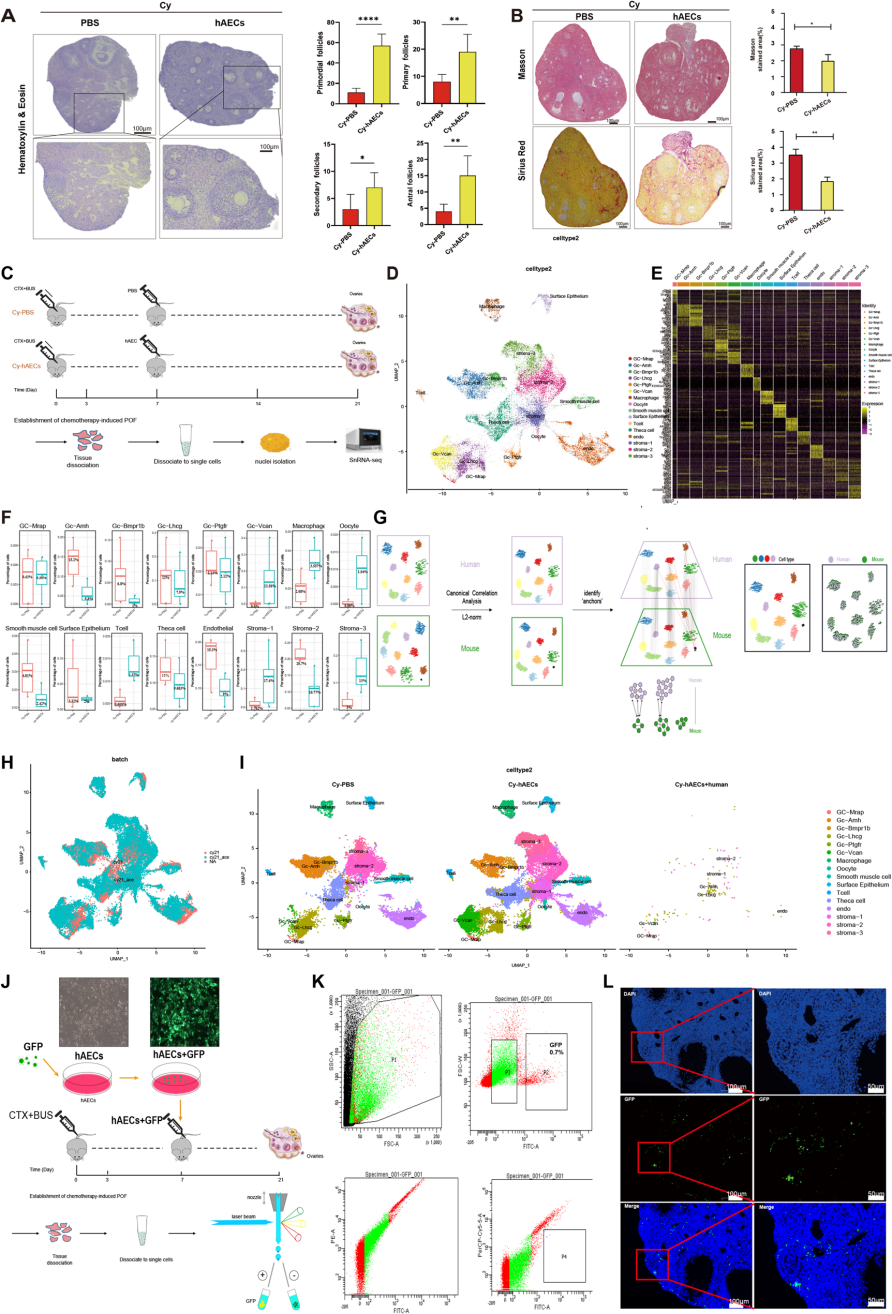
Effect of hAEC transplantation on the heterogeneity of ovarian cells. **A** HE-stained ovarian sections and quantitative analysis of follicle numbers in the Cy-PBS and Cy-hAEC groups. **B** Masson’s trichrome and Sirius red staining indicating collagen deposition and fibrosis in ovarian tissues. **C** Schematic and experimental timeline of the POI mouse model with hAEC transplantation. **D** UMAP visualization of snRNA-seq data from 43,420 ovarian cells across 16 samples, annotated by cell cluster. **E** Heatmap of cluster-specific marker genes, highlighting selected murine markers. **F** Bar plot showing the proportional distribution of predicted cell types within each cluster. **G** Schematic of a deep learning-based “projection model” designed to compare nuclear transcriptomic profiles between human and murine cells using human-specific gene expression. **H**–**I** Projection of human cell transcriptomes onto the murine ovarian cell UMAP embedding using the model. **J** Strategy for GFP-labeled hAECs using lentiviral transduction. **K** Flow cytometry isolation of GFP-positive hAECs from ovarian single-cell suspensions. **L** Immunofluorescence staining of GFP in ovarian sections after hAEC transplantation. Data are presented as mean ± SEM; *P < 0.05, **P < 0.01, ***P < 0.001, ****P < 0.0001. Scale bars: 50 μm and 100 μm

In previous study, hAEC transplantation promoted follicular development and partially improved fertility in a chemotherapy-induced POI mouse model [10], but the mechanism was not thoroughly explored. Consistent with prior findings, hAEC transplantation increased follicle count, promoted antral follicle development, and reduced ovarian fibrosis (Fig. 4A–B). To evaluate the impact of hAECs on ovarian composition, we conducted a comparative analysis of snRNA-seq data between Cy-hAECs and Cy-21 Day (Fig. 4C). We identified a median of 1,517 genes per individual per group (Supplementary File 2). hAEC transplantation increased heterogeneity across ovarian cell types, particularly among oocytes, macrophages, and T cells, alongside expansion of Vcan^+^ granulosa cell and stromal subpopulations Stroma-1 and Stroma-3 (Fig. 4D-F).

Although prior tracing study has confirmed hAEC migration and granulosa cell differentiation [11], the proportion of homing remained unclear. Here, we developed a “projection model” to map human hAEC transcriptomes onto a murine ovarian reference atlas (Fig. 4G). This approach revealed that grafted hAECs localized primarily to granulosa cell subtypes, including GC-Lhcg (90/143), GC-Mrap (11/143), GC-Amh (3/143), and GC-Vcan (2/143), as well as stromal cells-specifically Stroma-1 (29/143) and Stroma-2 (7/143) and endothelial cells (1/143) (Fig. 4H–I). Overall, homing hAECs accounted for approximately 0.5% (143/28,215) of total ovarian cells. We further validated these findings using GFP-labeled hAECs. Flow cytometry results showed that ~ 0.7% of ovarian cells were GFP⁺, consistent with projection model predictions (Fig. 4J-K). Immunofluorescent results displayed that GFP signals were predominantly localized in granulosa cells and interstitial regions (Fig. 4L).

To further investigate the therapeutic function of hAECs in POI, we integrated snRNA-seq data from Sham, Cy-PBS, and Cy-hAECs groups and assessed the expression of POI-related pathogenic genes previously reported by Chen et al. Compared to the Sham group, the Cy-PBS group showed downregulation of Bmp6, Brca2, Spidr, Nr5a1, and Lgr4; hAEC transplantation partially restored their expression. In contrast, FSHR and Ppm1b were upregulated in the Cy-PBS group but decreased after hAEC treatment (Supplementary Fig. 2). These results demonstrate that hAEC transplantation exerts broad therapeutic effects on chemotherapy-induced POI and regulates POI-associated gene expression.

### hAECs promote follicular development by restoring granulosa cell and oocytes communication

**Fig. 5:**
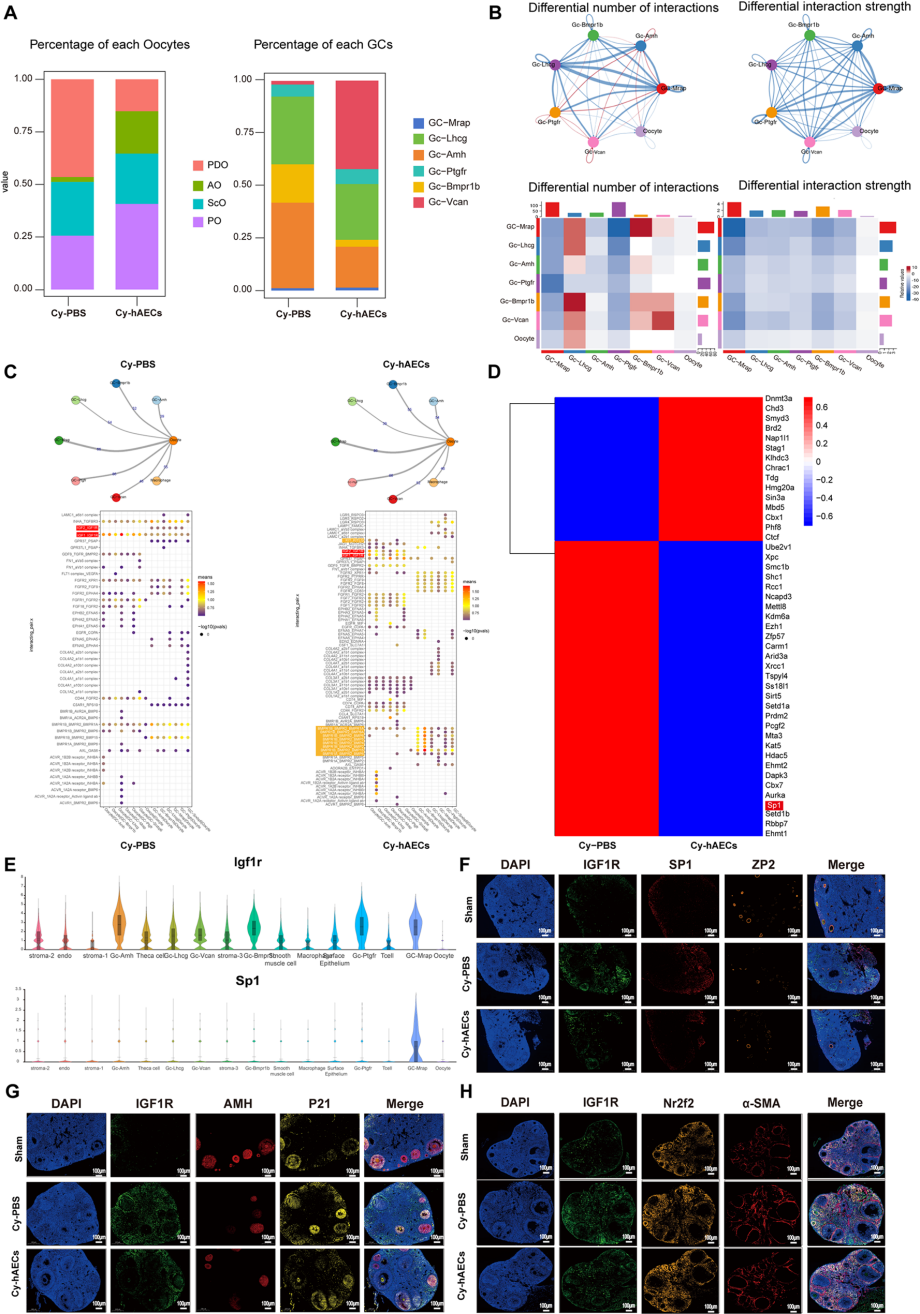
Effect of hAEC transplantation on follicular development. **A** Stacked bar plot showing the proportional distribution of oocyte and granulosa cell subclusters in Cy-PBS and Cy-hAECs groups. **B** Circle plot visualizing differential cell-cell communication between oocytes and granulosa cells. Red and blue edges indicate increased and decreased signaling, respectively, in Cy-hAECs compared to Cy-PBS. **C** Weights/Strength between granulosa cells and oocytes across groups. Dot plot displays the mean expression level and percentage of cells expressing selected ligand-receptor pairs, analyzed per sample group. **D** Effect of hAEC transplantation on the expression of transcription factors associated with follicular development in injured ovaries. **E** Violin plots showing expression levels of Igf1r and Sp1 across ovarian cell types. **F** Immunofluorescence staining of IGF1R/SP1/ZP2 in ovarian sections across different treatment groups. **G**-**H** Immunofluorescence staining of IGF1R/AMH/P21 and IGF1R/Nr2f2/α-SMA in ovarian sections among different treatment groups. Scale bars: 100 μm

To assess the impact of hAEC transplantation on folliculogenesis, we analyzed stage-specific changes in oocyte composition. hAEC transplantation significantly increased the proportion of antral oocytes (AO) and stimulated the activation of primordial oocytes into the primary stage. Meanwhile, they also enhanced granulosa cell (GC) heterogeneity (Fig. 5A). Given that chemotherapy disrupting granulosa cell and oocyte (OC) communication, we investigated whether hAEC transplantation could restore this communication. CellChat analysis revealed that hAECs significantly enhanced the number and strength of GC-OC signaling, including signals from GC-Amh and GC-ptgfr subpopulations to oocytes, as well as Kit-Kitlg signaling. Moreover, hAECs recovered the chemotherapy-induced hyperactivation of insulin-related pathways (Fig. 5B–C). In addition, hAECs modulated several transcription factors, including suppression of SP1, a regulator of primordial follicle activation. Transcriptomic profiling further revealed distinct spatial expression patterns, IGF1R was broadly expressed in granulosa cells and stromal compartments, while SP1 expression was predominantly restricted to the GC-Mrap subpopulation (Fig. 5D–E).

Immunofluorescence validation confirmed IGF1R upregulation in somatic cells of Cy-PBS ovaries, which was attenuated by hAEC transplantation. SP1 expression paralleled that of IGF1R across GC subtypes. Importantly, IGF1R overexpression correlated with granulosa cell senescence (P21 in AMH⁺ granulosa cells) and stromal fibrosis (α-SMA in Nr2f2⁺ stromal cells). hAEC transplantation effectively reduced both P21 and α-SMA expression (Fig. 5F–H). These results indicate that hAEC transplantation promotes follicular development, partially by regulating IGF1R-mediated signaling and by mitigating granulosa cell senescence and stromal fibrosis.

### hAECs repair ovarian function via IGFBP2-regulating IGF1R/Akt/mTOR signaling

**Fig. 6:**
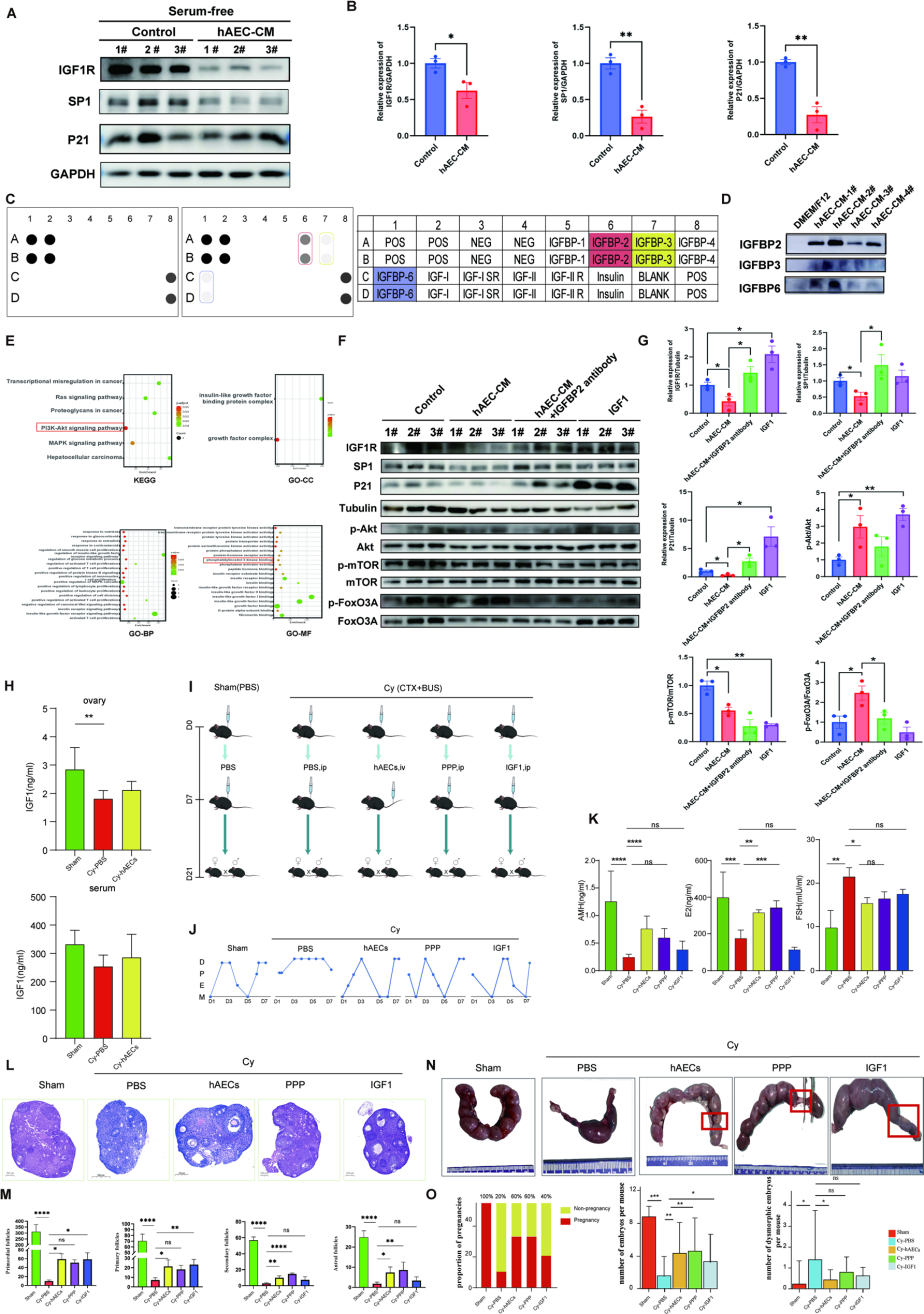
Targeted regulation of IGF1R improves ovarian function and fertility. **A**-**B** Western blot analysis of IGF1R, SP1, and P21 expression in KGN granulosa cells across treatment groups. **C** Human cytokine array profiling of IGF family proteins in hAEC-conditioned medium (hAEC-CM). **D** Western blot detection of IGFBP2, IGFBP3, and IGFBP6 in hAEC-CM from four biological replicates. **E** GO and KEGG enrichment analysis of downstream signaling pathways associated with IGFBPs identified in hAEC-CM. **F**-**G** Western blot detection of IGF1R/SP1/P21 and Akt/mTOR/FoxO3A pathway expression in control, hAEC-CM, hAEC-CM plus IGFBP2 antibody and IGF1-treated groups. **H** IGF1 concentrations in ovarian tissue and serum across experimental groups. **I** Schematic of the in vivo experimental design, including Sham, chemotherapy-injured (Cy), hAEC-transplanted (Cy-hAECs), IGF1R inhibitor-treated (Cy-PPP), and IGF1-supplemented (Cy-IGF1) groups. **J** Estrous cycle staging across treatment groups. **K** ELISA quantification of serum AMH, E2 and FSH levels. **L** Representative HE-stained ovarian sections from each group. **M** Quantification of primordial, primary, secondary, and mature follicles per ovary. **N** Isolation of uteri from pregnant mice prior to delivery. **O** Pregnancy rate, number of embryos per mouse, and proportion of abnormal embryos across groups (*n* = 5 mice per group). Data are presented as mean ± SEM; *P < 0.05, **P < 0.01, ***P < 0.001, ****P < 0.0001. Scale bars: 200 μm

Our previous cytokine array profiling identified insulin-like growth factor-binding proteins (IGFBPs) as key secretory components of hAECs targeting the IGF system. [12] To investigate the functional impact of hAEC-derived paracrine factors, we collected the conditioned medium of hAECs (hAEC-CM) and assessed its effects in serum free-induced the senescence of human granulosa cells (KGN). Western blot results showed that hAEC-CM significantly reduced IGF1R, SP1, and P21 expression (Fig. 6A–B). Further cytokine analysis confirmed abundant IGFBP2, IGFBP3, and IGFBP6 secretion across multiple hAEC samples, but not IGF1 (Fig. 6C–D). GO and KEGG analysis confirmed that the downstream signaling mainly focused on the PI3K/Akt pathway (Fig. 6E). Western blot analysis revealed that hAEC-CM suppressed the expression of IGF1R, SP1, and P21 in KGN cells under serum-free conditions, while promoting the phosphorylation of Akt and FoxO3A. Neutralization of IGFBP2 in hAEC-CM attenuated its inhibitory effects on IGF1R, SP1, and P21, and abolished hAEC-CM–mediated regulation of p-Akt/Akt and p-FoxO3A/FoxO3A ratios. In contrast, direct supplementation of IGF1 significantly upregulated p-Akt but also induced overexpression of IGF1R, leading to increased expression of cellular senescence-associated protein P21 (Fig. 6F–G). These findings indicate that hAECs could regulate IGF1R/Akt signaling in granulosa cells via secreting IGFBP2.

Circulating IGF1, which primarily originates from the liver, systemically regulates IGF1R activity. Our ELISA results showed that chemotherapy reduced IGF1 levels in both serum and ovarian lysates, and this reduction was not restored by hAEC transplantation (Fig. 6H). To further assess the therapeutic relevance, we designed in vivo intervention experiments, including hAEC treatment, IGF1R inhibitor (PPP) intervention, and recombinant IGF1 supplementation. The estrous cycle results indicated that all three intervention methods partially restored cyclicity in chemotherapy-induced POI mice (Fig. 6J). ELISA results showed that chemotherapy significantly decreased the levels of AMH and E2, and increased FSH in serum. Notably, hAEC transplantation increased AMH and E2 levels, and decreased FSH, PPP intervention markedly elevated the level of E2 (Fig. 6K). Morphological results displayed that hAEC transplantation increased follicle count across all stages, whereas PPP primarily expanded secondary and mature follicles, and IGF1 increased primordial and primary follicles (Fig. 6L–M). Furthermore, fertility analysis showed that all three treatments improved pregnancy rates and embryo yields, with hAECs exhibiting the best protection against embryonic abnormalities (Fig. 6N–O). Taken together, these results demonstrate that IGFBP2 secreted by hAECs can modulate the IGF1R/Akt/mTOR pathway in granulosa cells and partially mitigate their senescence. Furthermore, pharmacological inhibition of IGF1R may represent a promising therapeutic strategy for restoring ovarian function.

## Discussion

This study delineates the snRNA transcriptomic landscape of mouse ovaries following chemotherapy, capturing both acute injury (at Day 3 post-chemotherapy) and ovarian function remodeling (at Day 21 post-chemotherapy) phases. We defined distinct oocyte and granulosa cell subpopulations, as well as reconstructed their developmental trajectories. Beyond conventional categorization, we identified expanded clusters of surface epithelial and theca cells. Furthermore, we uncovered chemotherapy-induced disruptions in granulosa cells and oocytes communication, providing a molecular basis for follicular development disorder. Although hAEC transplantation could repair ovarian function in POI mouse model, its mechanisms remain unclear. Using a novel snRNA-seq-based “projection model”, we tracked transplanted hAECs in damaged ovaries and analyzed the homing efficiency. Meanwhile, hAEC transplantation promoted residual follicle development by restoring granulosa–oocyte communication, in which hAECs regulated the IGF1R/Akt pathway in granulosa cells by secreting IGFBP2.

The mechanisms underlying chemotherapy-induced POI primarily involve three pathological processes, including excessive activation of primordial follicles leading to depletion of the ovarian reserve, DNA damage-triggered apoptosis and cell death, and injury to microvasculature and stromal compartments [22]. Based on existing evidence, researchers have proposed an integrated hypothesis that accounts for both acute and delayed ovarian injury following chemotherapy [23]. Our recent study further supports this model by demonstrating that a single course of combination chemotherapy acutely suppresses granulosa cell proliferation and triggers apoptosis, whereas the chronic phase of ovarian damage is characterized by persistent follicle loss, cellular pyroptosis, and inflammation-associated fibrosis [24]. Thus, chemotherapy-induced POI is a multifaceted process involving both germ and somatic cell compartments within the ovary. A systematic analysis of post-chemotherapy ovarian cell composition and follicular dynamics is therefore essential to elucidate injury mechanisms and identify potential therapeutic targets.

Our study systematically analyzed the impact of chemotherapy on ovarian cell composition and provided a clear delineation between oocytes and somatic cells. We observed that chemotherapy acutely reduced the proportion of granulosa cells and oocytes, directly linked to follicular depletion. Subsequently, impaired follicular development emerged as a key pathological hallmark during the late phase of ovarian injury. In addition, study further showed that chemotherapy not only disrupted follicular integrity but also induced dysregulation of ovarian immune homeostasis and somatic cell distribution. In the aging mouse ovary, accumulation of immune cells and decreased collagenase expression in the stroma collectively promote ovarian inflammation and fibrosis [25]. These insights underscore that ovarian protection strategies during early chemotherapy should prioritize rescuing follicular cells, whereas post-chemotherapy ovarian recovery ought to focus on restoring the microenvironment and supporting follicular development.

Stem cell transplantation has emerged as a promising therapeutic strategy for POI, marking a significant transition from basic research toward clinical application. However, the homing efficiency of transplanted cells remains incompletely understood. As early as 2013, our team demonstrated that a few hAECs could migrate into injured ovaries and differentiate into granulosa cells expressing FSHR [11]. Subsequent studies corroborated these findings, showing that hAECs administered via tail vein injection primarily localized to the interstitial regions of damaged ovaries [26]. A recent report further revealed that intravenously transplanted hAECs could infiltrate the granulosa cell (GC) layer of follicles and remain detectable 28 days after transplantation [27].

In the present study, we employed advanced deep learning approaches to develop a “projection model” capable of assessing transcriptional similarity between individual human and murine cells at single-nucleus resolution. Using this model, we observed that only a small number of hAECs engrafted, predominantly into granulosa and stromal cell populations rather than oocytes, which aligns with prior histological findings. Quantification based on this model revealed that homing hAECs account for approximately 0.5% of total ovarian cells in the transplantation group. Meanwhile, this projection model was further validated through GFP-based cell tracking, which confirmed a homing efficiency of 0.7%. These findings offer important insights into the homing behavior of hAECs within the damaged ovarian microenvironment. Importantly, it also indicated direct homing of hAECs constitutes only a minor component of ovarian repair, whereas robust paracrine function plays a central role in hAECs-mediated restoration of ovarian function. Furthermore, this study highlights the safety profile of hAEC transplantation, supporting its potential as a therapeutic strategy for patients with POI.

Indeed, growing evidence suggest that trophic factors secreted by stem cells can effectively ameliorate chemotherapy-induced ovarian damage and decelerate age-related fertility decline [28]. The insulin-like growth factor 1 receptor (IGF1R) is a cell-surface receptor tyrosine kinase that, upon binding its ligands IGF1 and IGF2, activates the downstream PI3K/Akt pathway to stimulate cell proliferation, differentiation, migration, and survival [29]. Within the ovary, IGF1 and its receptor IGF1R are selectively expressed in granulosa cells of developing follicles and stromal cells. They play critical roles in FSH-stimulated steroidogenesis [30] and in the differentiation of pre-antral into pre-ovulatory granulosa cells, a process dependent on IGF1R activity and subsequent Akt activation [31]. Numerous studies underscore the importance of IGF1R signaling in maintaining stemness and improving the efficacy of stem cell-based therapies [32]. A study demonstrated that intravenous injection of embryonic stem cell-derived mesenchymal stem cells was shown to alleviate colitis in mice by elevating circulating IGF1 levels [33]. Similarly, IGFBP-3 secreted by human amniotic mesenchymal stem cells attenuated liver fibrosis in mice by suppressing hepatic stellate cell activation via inhibition of the Wnt/β-catenin pathway [34]. However, in the process of chronic injury repair, IGF1R is generally considered an aging promoting factor that accelerates tissue fibrosis [35]. In this study, we observed that the level of IGF1 in ovaries significantly decreased after chemotherapy; however, transplantation of hAECs did not restore IGF1 level. Previous study has identified several bioactive paracrine factors, including IGFBPs in hAEC-CM . Here, we confirmed that hAECs predominantly secrete IGFBP2, IGFBP3, and IGFBP6, but not IGF1. Importantly, we discovered that aberrant IGF1R expression accelerates granulosa cell senescence and stromal fibrosis. Transplantation of hAECs reversed this IGF1R dysregulation and counteracted granulosa cell aging. This protective effect is mediated through hAEC-secreted IGFBP2, which acts by regulating the PI3K/Akt pathway.

This study has certain limitations that should be acknowledged. First, the present snRNA-seq data elucidate transcriptomic alterations in key ovarian cell types, particularly within granulosa cells and during oocyte development, as well as changes in cellular communication. To fully understand the folliculogenesis microenvironment, future studies should complement these findings by defining the transcriptomic profiles of stromal and immune cells. Furthermore, while *n* = 3 sample size is common in exploratory snRNA-seq studies, it may constrain the statistical power to detect more subtle changes or rare cell populations. Second, this study currently lacks direct evidence for a physical interaction between IGFBP2 and IGF1R, as well as IGFBP2-mediated transcriptional regulation of the IGF1R/Akt/mTOR axis. Thus, future research should focus on the time- and dose-dependent effects of IGFBP2 on downstream signaling, which is crucial to fully elucidate the mechanism underlying granulosa cell functional recovery. Third, current fertility assessments primarily relies on pregnancy rates and embryo number, these metrics do not fully capture the functional endpoint of fertility. Future studies should focus on ovarian functional endpoints, including litter size and offspring health. Fourth, although both IGF1R inhibition and IGF1 supplementation have been shown to enhance follicular development at distinct stages in animal models, their precise mechanisms remain incompletely understood. Future work should focus on stage-specific IGF1R regulation or dose-response analyses in granulosa cells from developing follicles to clarify these dynamics. Additionally, the absence of comprehensive pharmacological validation in our in vivo IGF1R inhibitor experiments limits a detailed assessment of the inhibitor’s efficacy and specificity within this POI model.

## Conclusions

Through establishing a chemotherapy-induced POI mouse model, we clarified the dynamic changes in ovarian cell composition and identified single-cell transcriptomic alterations in granulosa cell and oocytes, further analysis revealed late-phase disruption of granulosa cell-oocyte communication, a mechanism likely contributing to impaired follicular development. Notably, hAEC transplantation significantly increased cellular heterogeneity within granulosa and stromal compartments. Moreover, hAECs restored intercellular crosstalk between granulosa cells and oocytes and promoted follicular development partially through IGFBP2-regulating IGF1R/Akt signaling pathway in granulosa cells. These findings provide important insights into chemotherapy-induced POI and establish a valuable resource for developing hAEC-based therapeutic strategies for premature ovarian insufficiency.

## Supplementary Information

Supplementary Material 1.

Supplementary Material 2. Supplementary Fig. 1. Characterization of cultured hAECs. (A) Morphology of cultured hAECs observed by light microscopy. Scale bar: 100 μm. (B) Flow cytometry analysis of surface markers (SSEA4, CD324, CD146, HLADR) expressed on hAECs. (C) Representative immunofluorescence images depicting the expression of epithelial markers (CK18 and CK7), the stem cell marker OCT4, and mesenchymal markers (N‑cadherin and Vimentin) in hAECs. Scale bars: 25 μm.

Supplementary Material 3. Supplementary Fig. 2. Impact of hAEC transplantation on the expression of POI-related pathogenic genes.

Supplementary Material 4.

Supplementary Material 5.

Supplementary Material 6.

## Data Availability

The data reported in this paper have been deposited in the OMIX, China National Center for Bioinformation/Beijing Institute of Genomics, Chinese Academy of Sciences (https://ngdc.cncb.ac.cn/omix: accession no.OMIX015442).

## Abbreviations

POI: Premature ovarian insufficiency
hAECs: Human amniotic epithelial cells
snRNA-seq: single-nucleus RNA sequencing
HRT: Hormone replacement therapy
PBS: Phosphate-buffered saline
EGF: Epidermal growth factor
PFA: Paraformaldehyde
PCA: Principal component analysis
UMAP: Uniform Manifold Approximation and Projection
DEGs: Differentially expressed genes
TEM: Transmission electron microscopy
HE: Hematoxylin and eosin
PSR: Picric acid–Sirius red
BSA: Bovine serum albumin
hAEC-CM: Conditioned medium of human amniotic epithelial cells
E2: Estradiol
FSH: Follicle-stimulating hormone
AMH: Anti-Müllerian hormone
CTX: Cyclophosphamide
PPP: Picropodophyllin
GC: Granulosa cell
PGGC: Progenitor GC
PLGC: Proliferative GC
CGC: Cumulus GC
MGC: Mural GC
PDO: Primordial oocyte
PO: Primary oocyte
ScO: Secondary oocyte
AO: Antral oocyte
OC-GC: Oocyte-granulosa cell
IGFBPs: Insulin-like growth factor-binding proteins
