# Supplementary figures and images for: hAECs restore follicular development in premature ovarian insufficiency via IGFBP2/IGF1R-mediated intercellular communication
Source: Stem Cell Res Ther. 2026 May 18;17:246. doi: 10.1186/s13287-026-05064-8 (PMC13352713; doi:10.1186/s13287-026-05064-8)

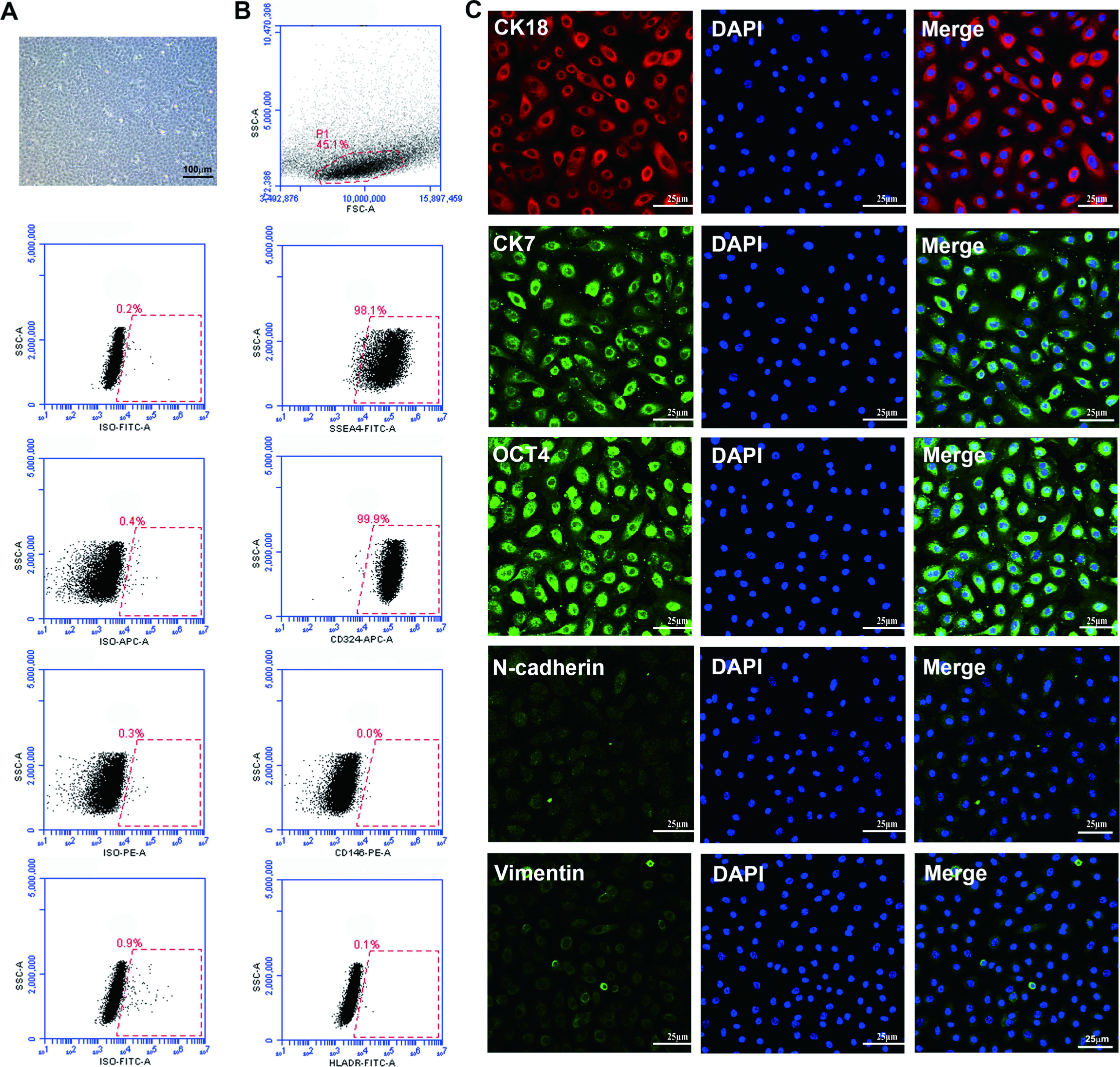

Supplement: Supplementary file 2 — Supplementary Material 2. Supplementary Fig. 1. Characterization of cultured hAECs. (A) Morphology of cultured hAECs observed by light microscopy. Scale bar: 100 μm. (B) Flow cytometry analysis of surface markers (SSEA4, CD324, CD146, HLADR) expressed on hAECs. (C) Representative immunofluorescence images depicting the expression of epithelial markers (CK18 and CK7), the stem cell marker OCT4, and mesenchymal markers (N‑cadherin and Vimentin) in hAECs. Scale bars: 25 μm. [file 13287_2026_5064_MOESM2_ESM.tif]

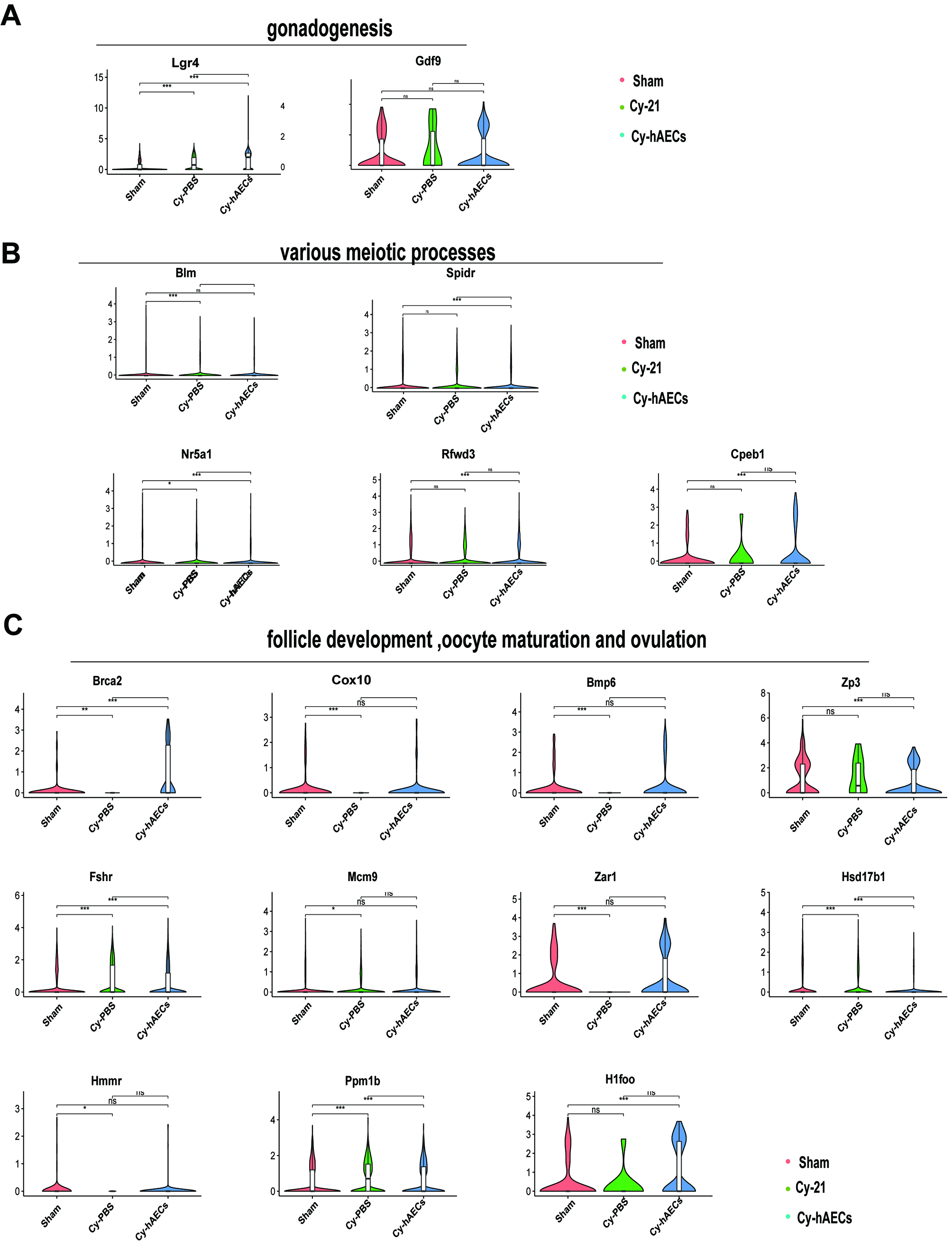

Supplement: Supplementary file 3 — Supplementary Material 3. Supplementary Fig. 2. Impact of hAEC transplantation on the expression of POI-related pathogenic genes. [file 13287_2026_5064_MOESM3_ESM.tif]
