# Supplementary material for: hAECs restore follicular development in premature ovarian insufficiency via IGFBP2/IGF1R-mediated intercellular communication
Source: Stem Cell Res Ther. 2026 May 18;17:246. doi: 10.1186/s13287-026-05064-8 (PMC13352713; doi:10.1186/s13287-026-05064-8)
Supplement: Supplementary file 6 — Supplementary Material 6. [file 13287_2026_5064_MOESM6_ESM.pdf]

Supporting Information

hAECs Restore Follicular Development in Premature Ovarian Insufficiency via IGFBP2/IGF1R-Mediated Intercellular Communication

Wenjiao Cao[1#], Lu Shen[2#], Qinyu Zhang[1], Yating Huang[1], Junyan Sun[1], Zixin Cheng[1], Jing Xu[3], Qiuwan Zhang[1*], Dongmei Lai[1*]

[1] International Peace Maternity and Child Health Hospital, Shanghai Key Laboratory of Embryo Original Diseases, School of Medicine, Shanghai Jiao Tong University, Shanghai, 200030, China

[2] Bio-X Institutes, Key Laboratory for the Genetics of Developmental and Neuropsychiatric Disorders, Ministry of Education, Shanghai Jiao Tong University, Shanghai, 200030, China.

[3] School of Biomedical Engineering, Shanghai Jiao Tong University, Shanghai, 200030, China

*Corresponding Author:

Dongmei Lai, MD, PhD

E-mail: laidongmei@hotmail.com

Qiuwan Zhang, PhD

E-mail: zhangqiuwan@163.com

145, Guang-Yuan Road, Shanghai 200030, P. R. China,

Tel: 86-21-64070434

Fax: +86-21-64074642

The original, uncropped Western blot images corresponding to Figure 6 are shown below. Protein molecular weight markers (kDa) are indicated. Areas within red boxes represent the cropped regions used in the Figure 6.

Figure 6A:

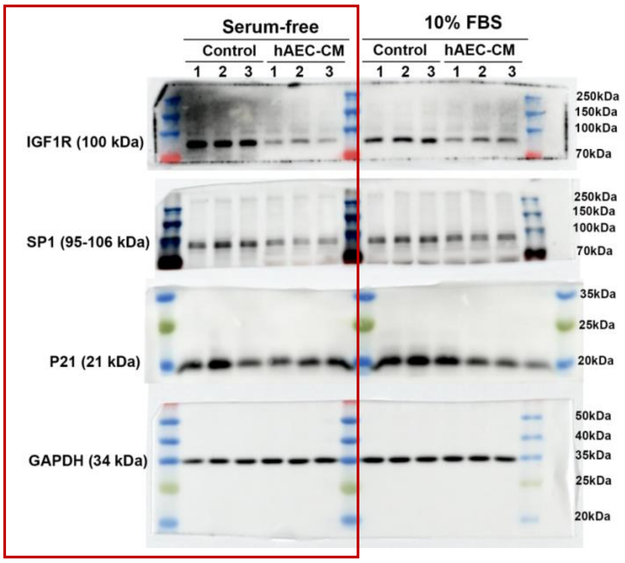

Figure 6D:

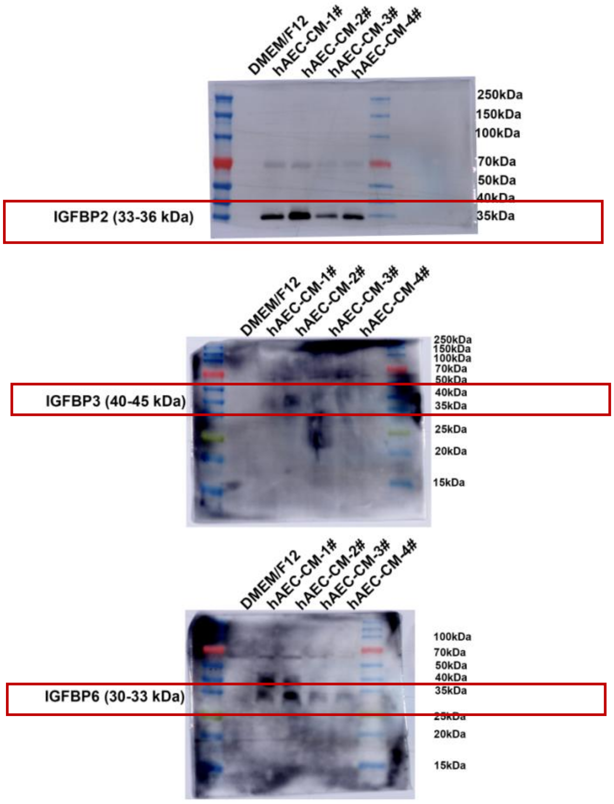

Figure 6F:

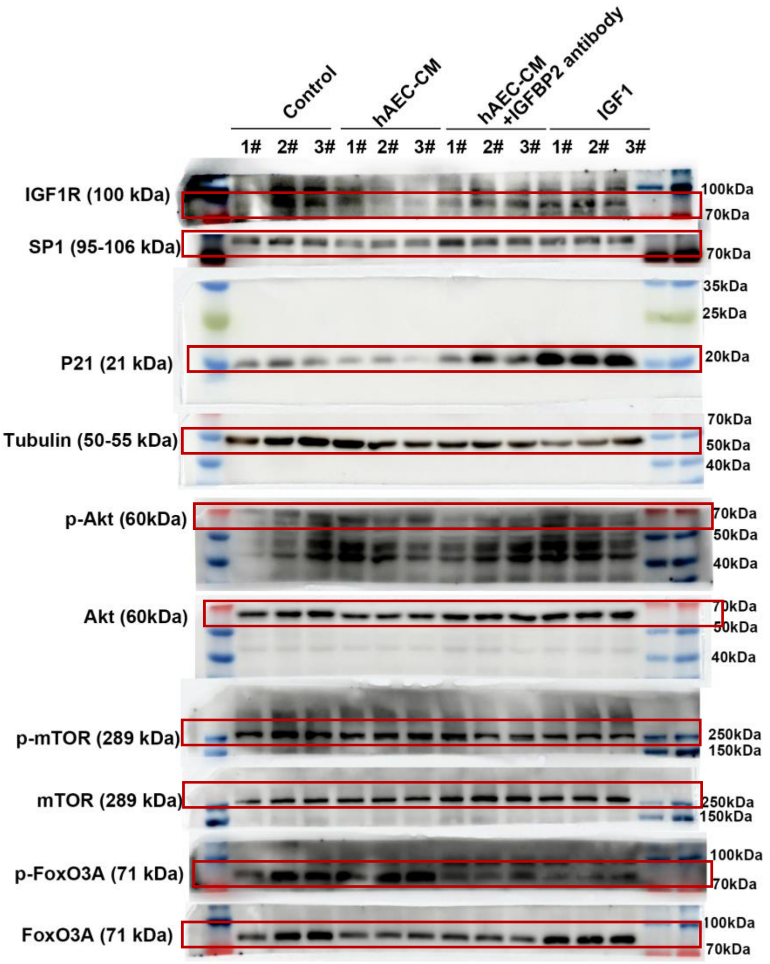
Control
hAEC-CM
hAEC-CM +IGFBP2 antibody
IGF1
1# 2# 3# 1# 2# 3# 1# 2# 3# 1# 2# 3#
IGF1R (100 kDa)
SP1 (95-106 kDa)
P21 (21 kDa)
Tubulin (50-55 kDa)
p-Akt (60kDa)
Akt (60kDa)
p-mTOR (289 kDa)
mTOR (289 kDa)
p-FoxO3A (71 kDa)
FoxO3A (71 kDa)
100kDa
70kDa
70kDa
35kDa
25kDa
20kDa
70kDa
50kDa
40kDa
70kDa
50kDa
40kDa
70kDa
50kDa
40kDa
250kDa
150kDa
250kDa
150kDa
100kDa
70kDa
100kDa
70kDa
